# Enzymatic hydrolysis of the gelatinous layer in tension wood of *Salix* varieties as a measure of accessible cellulose for biofuels

**DOI:** 10.1186/s13068-021-01983-1

**Published:** 2021-06-22

**Authors:** Jie Gao, Mohamed Jebrane, Nasko Terziev, Geoffrey Daniel

**Affiliations:** Department of Forest Biomaterials and Technology/Wood Science, Swedish University of Agricultural Sciences, Box 7008, 750 07 Uppsala, Sweden

**Keywords:** *Salix viminalis*, Bioenergy crops, Enzymatic saccharification, Lignocellulose biofuels, Biomass recalcitrance

## Abstract

**Background:**

*Salix* (willow) species represent an important source of bioenergy and offer great potential for producing biofuels. *Salix* spp. like many hardwoods, produce tension wood (TW) characterized by special fibres (G-fibres) that produce a cellulose-rich lignin-free gelatinous (G) layer on the inner fibre cell wall. Presence of increased amounts of TW and G-fibres represents an increased source of cellulose. In the present study, the presence of TW in whole stems of different *Salix* varieties was characterized (i.e., physical measurements, histochemistry, image analysis, and microscopy) as a possible marker for the availability of freely available cellulose and potential for releasing d-glucose. Stem cross sections from different *Salix* varieties (Tora, Björn) were characterized for TW, and subjected to cellulase hydrolysis with the free d-glucose produced determined using a glucose oxidase/peroxidase (GOPOD) assay. Effect of cellulase on the cross sections and progressive hydrolysis of the G-layer was followed using light microscopy after staining and scanning electron microscopy (SEM).

**Results:**

Tension wood fibres with G-layers were developed multilaterally in all stems studied. *Salix* TW from varieties Tora and Björn showed fibre G-layers were non-lignified with variable thickness. Results showed: (i) Differences in total % TW at different stem heights; (ii) that using a 3-day incubation period at 50 °C, the G-layers could be hydrolyzed with no apparent ultrastructural effects on lignified secondary cell wall layers and middle lamellae of other cell elements; and (iii) that by correlating the amount of d-glucose produced from cross sections at different stem heights together with total % TW and density, an estimate of the total free d-glucose in stems can be derived and compared between varieties. These values were used together with a literature value (45%) for estimating the contribution played by G-layer cellulose to the total cellulose content.

**Conclusions:**

The stem section-enzyme method developed provides a viable approach to compare different *Salix* varieties ability to produce TW and thus freely available d-glucose for fermentation and biofuel production. The use of *Salix* stem cross sections rather than comminuted biomass allows direct correlation between tissue- and cell types with d-glucose release. Results allowed correlation between % TW in cross sections and entire *Salix* stems with d-glucose production from digested G-layers. Results further emphasize the importance of TW and G-fibre cellulose as an important marker for enhanced d-glucose release in *Salix* varieties.

## Background

The use of *Salix* for bioenergy (electrical) and heat energy production has a long history [1]. More recent interest has, however, been focused on the possible use of *Salix* feedstocks for production of transportation fuels particularly bioethanol and biogas [2–6]. Features that make *Salix* interesting for biofuel include its rapid growth, the possibility for growth on barren and poor soil, relatively low lignin content in contrast to other wood species, and its high annual volume production per hectare [7].

An important parameter for selection of any plant/wood species for biofuel production is based on the lignocellulose material recalcitrance, which is related to the chemistry and relation between the major biomass polymers cellulose, hemicelluloses and lignin. In wood species, the cellulose content normally resides within the range 40–50% in both hardwoods and softwoods, while lignin, and hemicelluloses tend to be inversely correlated [8]. In woody lignocellulose, the main polymers are interrelated at molecular and ultrastructural levels with cellulose primarily in contact with the hemicelluloses that are primarily bound to lignin [9], the arrangement the ultimate supramolecular basis for recalcitrance. Recalcitrance in bioenergy plants is also related to total lignin content and is why low lignin containing and extractive free plant species like fast-growing *Salix* and its close relative Poplar have shown to be the most attractive options for biofuels. During bioethanol production, cellulose in the biomass is usually converted to fermentable sugars through thermal- or chemical pretreatments. This is followed by enzymatic hydrolysis with enzymes (i.e., cellulases), the glucose simultaneously converted by yeast or conducted in a separate process [1]. Thus, for bioethanol production, the most important and limiting factors will be the total cellulose content and its accessibility in the lignocellulose biomass structure for enzymatic hydrolysis.

*Salix* is a member of the Salicaceae family, and numerous varieties have been selected and commercially developed over the years for their rapid growth and volume production per hectare [10–12]. *Salix* spp. has a diffuse to semi-ring-porous wood anatomy characterized by the presence of fibres, parenchyma cells and radially orientated vessels (often clustered in 2–3 s) in different ratios depending on species and variety. Like many hardwoods, *Salix* spp. develop abnormal (i.e., reaction) wood under particular circumstances during which a characteristic tissue and cellular type known as tension wood (TW) containing gelatinous fibres (i.e., G-fibres) are produced [13]. In certain hardwoods (e.g., poplar, willow, oak, birch) this reaction is particularly prominent in tree branches with TW produced on the upper side of branches, the response helping to maintain the branch in a perpendicular orientation to the axial stem [14]. TW also occurs in young developing stems (e.g., 1–4-year-old plants) of *Salix* and its close relative Poplar [15, 16]. Studies have further shown enhanced TW development in environments, where stems are exposed to strong adverse winds suggesting differences in both gravimetric responses and possibly genetic differences [15, 17].

Anatomically, TW is composed of characteristic G-fibres with the most characteristic type producing an additional cellulose-rich layer, known as the gelatinous layer (G-layer) on the lumen side of the secondary cell walls of fibres [14, 18, 19]. G-fibres with gelatinous layers are cellulose-rich and in contrast to the other cell wall layers forming the outer secondary cell wall, the G-layer either lacks or has very low lignin levels in many hardwood species [14, 20]. Early reports suggested a cellulose (as d-glucose) content as high as 98.5% [20] in *Populus tremula*, although more recent estimates of isolated G-layers from *P. alba* give values in the region of 75–78% together with matrix materials of hemicelluloses [21, 22]. The cellulose in the G-layer is reported as highly crystalline with X-ray diffraction showing 60% crystallinity with monoclinic 1β cellulose crystals [23]. Various microscopy approaches have further shown G-layers in different hardwoods to consist morphologically of microfibrils and microfibril aggregates (macrofibrils; ranging from 10 to 60 nm) held in an interconnected honeycomb gel construction, with mesopores of the order 2–50 nm [16, 24–26] alternatively 6–12 nm [27]. All these morphological aspects are consistent with free access to enzymatic attack from the fibre cell lumen when exposed.

Recent studies with *Salix* have shown the presence of TW in stems processed for biofuel production using cellulase hydrolysis to confer higher bioethanol yields [15]. While some debate exists on what is more important, the total cellulose content or cellulose accessibility or a combination of both [28], the presence of TW can be recognized as an important marker for biofuel potential. *Salix* varieties prone to producing large amounts of TW could, therefore, be seen as a favorable option for biofuel production. When assessing different varieties of *Salix* for biotechnology and biopolymer yields, it is normal practice to comminute entire stems with bark by chipping/refining for traditional wet chemical analysis or more recently use high-throughput chemical screening, both approaches involving random sampling of the total initial *Salix* variant biomass [29]. The former approach enables the processing and comparison of large number of species/varieties and reflects industrial situations. However, while these approaches overcome the problems of processing large numbers of samples, only very small amounts of biomass are used (often mgs). Thus, any differences in tissue type in stems processed under these conditions would tend to be lost. For example, stems showing TW also attributes generally show opposite-(OW, i.e., the wood formed on the opposite side of the TW tissue) and normal wood (NW, i.e., wood without reaction tissue) [14, 18] and thus bulk chemical analysis may only show small differences in polymeric chemical composition without some form of pre-separation. Similarly, analysis of only discrete regions from stems as frequently done for basal stem regions is generally argued as not representative of the situation along the stem. Thus even if a *Salix* variety was shown to have significant TW with increased cellulose content and accessibility at basal height above ground, this may not necessarily be related to the entire stem and *Salix* variant genotype or phenotypic response.

In the present study, we developed a method to assessed entire stem cross sections from different *Salix* varieties and applied a commercial cellulase to determine accessible cellulose as an indirect method to quantify the amount of TW tissue and readily accessible d-glucose. Thus, the higher the d-glucose concentration determined, the greater the amounts of TW fibres and accessible d-glucose and the most promising varieties for accessible sugar and bioethanol production.

## Results and discussion

The physical dimensions of 2-year growth of the *Salix* varieties used for studies varied from ca. 200 to 400 cm in height (Tables 1, 2, 3) and between 1.85 cm (Jorr) and 2.83 cm (Tora) at 40 cm above the ground to 1.05 cm (Tora) at 400 cm in diameter (debarked samples, Table 1). Only 2-year growth was studied with the majority of the *Salix* varieties ca. 60–100 cm taller including first year growth.

**Table 1:** Diameter of debarked *Salix* varieties along the stem

Stem height (cm)	Diameter (cm) of different stems				
	Tora	Tora + F	Björn	Loden	Jorr
400	1.05				
360	1.35	1.33	1.36		
320	1.53	1.49	1.62		
280	1.69	1.65	1.80	1.08	
240	1.91	1.83	1.91	1.22	
200	2.04	1.98	2.06	1.20	1.36
160	2.20	2.12	2.20	1.38	1.47
120	2.35	2.24	2.35	1.52	1.58
80	2.52	2.42	2.53	1.69	1.72
40	2.83		2.73	1.90	1.85
0				2.35	1.86

**Table 2:** Total d-glucose measured as mg/g per kg/m^3^ dry wt along the stem of *Salix* variety Tora

Stem height (cm)	Tora		
	Density (kg/m^3^)	d-Glucose amount (mg/g)	d-Glucose amount (kg/m^3^)
400	450.5	155.7	69.7
360	454.4	124.4	56.5
320	455.4	82.2	37.4
280	452.6	95.8	43.4
240	459.3	81.9	37.6
200	462.3	67.9	31.4
160	465.7	64.0	29.8
120	473.0	60.2	28.5
80	491.7	49.7	24.4
40	522.4	35.0	18.3
Average	468.7		37.7

**Table 3:** Total d-glucose measured as mg/g per kg/m^3^ dry wt along the stem of *Salix* variety Björn

Stem height (cm)	Björn		
	Density (kg/m^3^)	d-Glucose amount (mg/g)	d-Glucose amount (kg/m^3^)
360	451.3	117.9	53.3
320	467.0	112.8	52.8
280	469.9	111.2	52.2
240	464.6	88.9	41.3
200	455.8	89.0	40.6
160	461.3	85.9	39.7
120	473.6	87.8	41.7
80	490.9	89.2	43.7
40	520.8	61.8	32.2
Average	472.8		44.2

In the present work, emphasis was placed on Tora and Björn which are commercial *Salix* varieties. Tora showed the tallest stems with only Björn having slightly thicker stems (with or without bark) in 6 points measured along the stem axis (Tables 1, 2, 3). All physical measurements were made on debarked samples. Ten cm stem cylinders were used for section analyses of TW and the enzymatic and staining studies. For further comparison, a wood sample taken from an 8-year-old tree of *S. viminalis* growing locally in Uppsala, Sweden was also included in the study. All enzyme studies were performed on debarked samples.

Macerated *Salix* tension-, normal- and opposite wood fibres from 1.0 to 4.5 cm wide stems (or 2-year-old stems) have an average length of ca. 1.0 mm. With our FiberMaster analysis of stems, 99% of the length weighted fibre length in *S. viminalis* was less than 1.5 mm and 96% less than 1.0 mm (unpublished observations). Thus, the thickness of the serial sections made (ca. 30 μm) was well within the average range of full fibre length allowing free access of the enzyme preparation into the fibre lumina (i.e., to avoid the presence of fibres with closed ends).

### Estimation of tension wood in *Salix* stem cross sections using image analysis

**Fig. 1:**
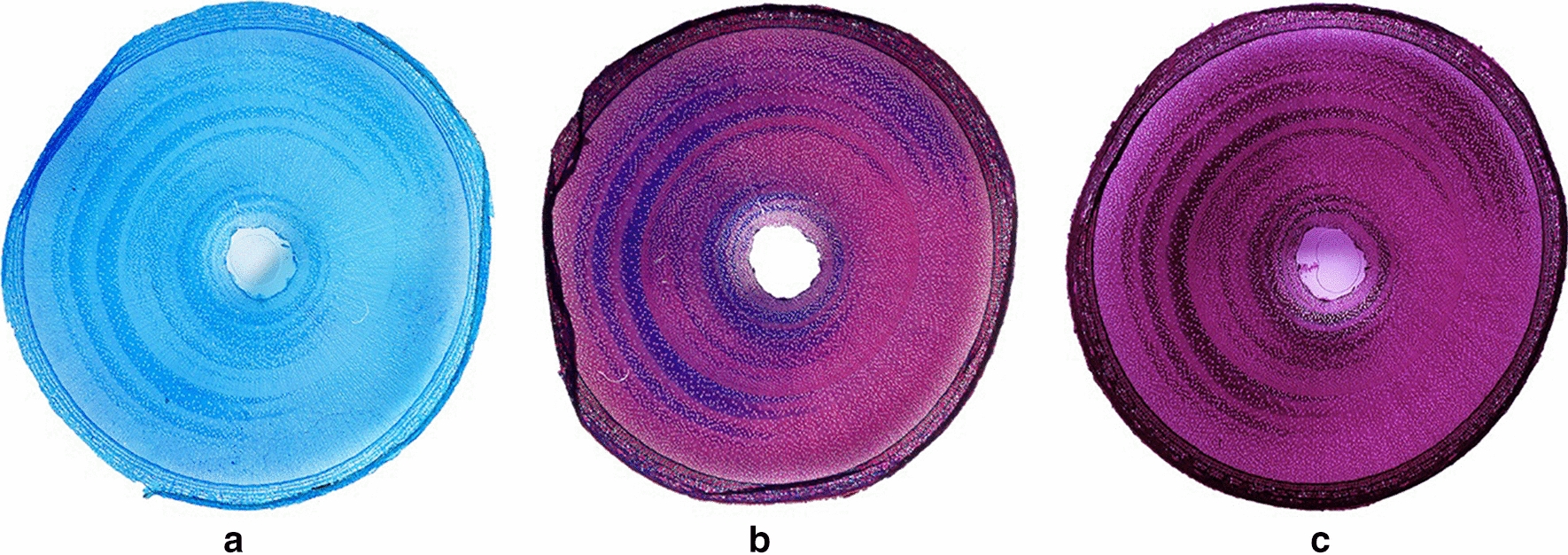
Example of *Salix* serial cross sections stained with astra blue (**a**), or double stained with either astra blue/safranin (**b**) or chlorazol black E/safranin (**c**)

**Fig. 2:**
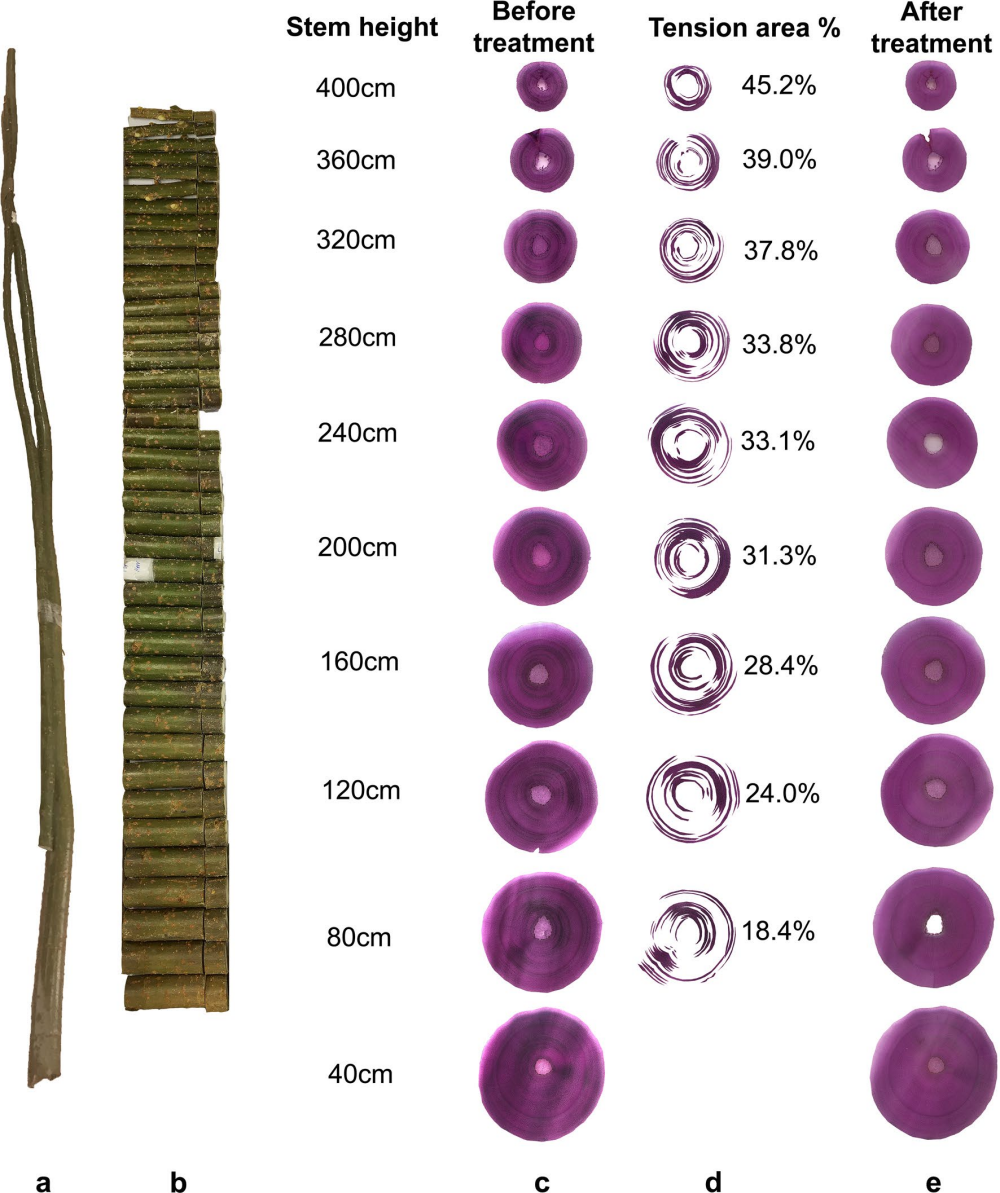
Overview of Tora stem (**a**), sawn cylinders (**b**, 10 cm long), debarked cross sections from different heights before, and after 3-day cellulase treatment and staining with chlorazol black E and safranin (**c** and **e**). Quantification of tension wood area (as %, **d**) using Photoshop

**Fig. 3:**
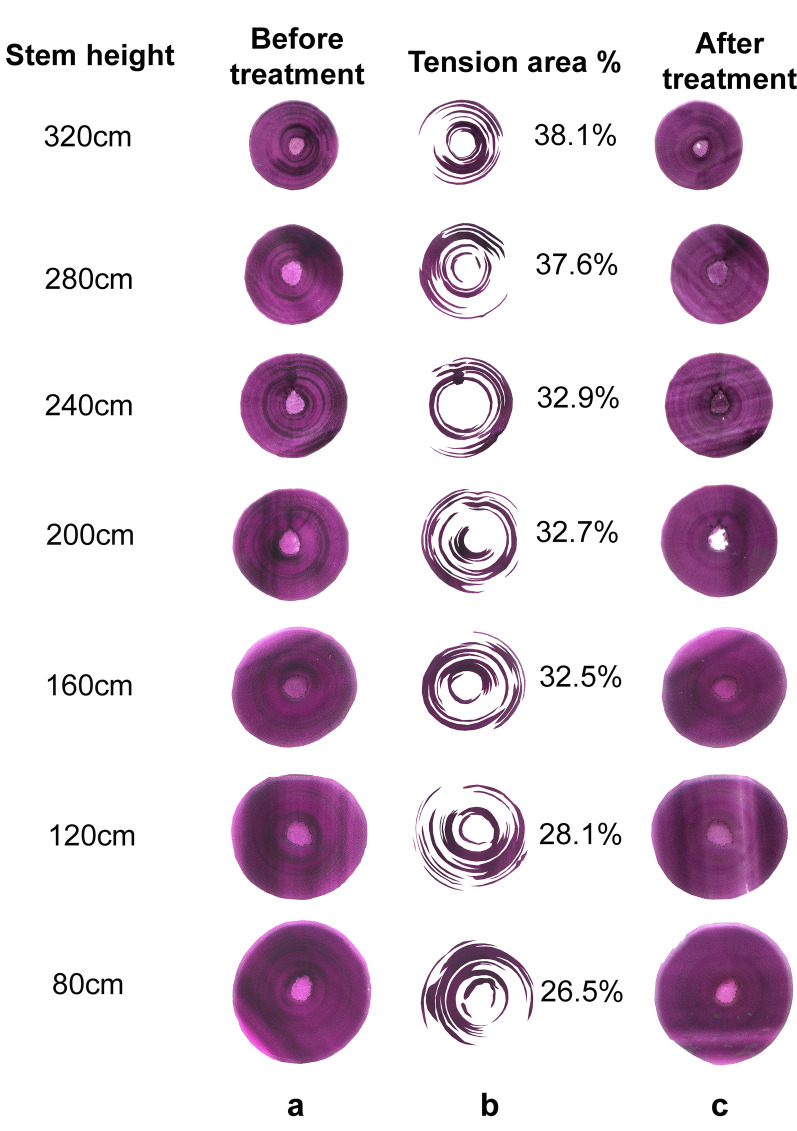
Overview of Björn stem debarked cross sections (**a**) from different heights before, and after 3-day cellulase treatment and double staining with chlorazol black E and safranin (**c**). Quantification of tension wood area (as %, **b**) using Photoshop

Presence of TW in cross sections of unstained *Salix* stems was visible prior to staining as cellular regions with glistening shiny appearance. For improved definition, however, three staining approaches were assessed initially including astra blue (single staining) and double staining with either safranin/astra blue and safranin/chlorazol black E (see “Materials and methods”; Fig. 1). Of the three approaches, safranin/chlorazol black E was chosen for analysis of TW tissue area as it provided improved definition for quantification when scanning entire cross sections. TW was present in *Salix* stem cross sections as discrete bands of distinct purple/black staining (Figs. 1, 2, 3) and as single fibres diffusely scattered in both the early- and latewood growth rings of all stems of Tora, Björn, Loden and Jorr. Only the TW bands were possible to estimate quantitatively. Light microscopy confirmed TW bands and presence of wood fibres with G-layers (G-fibres). Distribution of TW was not proportionate to first and second growth ring thickness and showed variability with some TW developing bands longer than half the stem circumference (Figs. 1, 2, 3). Differences in total TW were evident in cross sections and varied with stem height/diameter and between *Salix* varieties and in Tora ranged from ca. 18% at 80 cm to ca. 45% at 400 cm with corresponding values in Björn ca. 27% (80 cm) and ca. 38% (320 cm) for similar heights (Figs. 2, 3). The percentage area of TW estimated in cross sections was used to estimate the volume of TW in 40 cm cylinders cut from the stems (Fig. 2 (left), Tables 2, 3) and together to estimate TW in entire stems. In both *Salix* varieties, the amount of TW calculated from cross sections as a percentage of actual cylinder volume increased with height as the total area of cross sections/volume of cylinders decreased (Figs. 2, 3).

Since areas of TW were frequently present on opposing sides of the same stem cross section (Figs. 2, 3), the induction of TW tissue was not unilateral and restricted from to one side as seen with TW in branches of hardwoods but rather multilateral (Figs. 2c, 3c). Thus the presence of opposite and normal wood was not possible to truly differentiate.

### Density

The 2-year *Salix* samples examined showed a typical semi-diffuse anatomical structure with radially orientated vessels, fibres and parenchyma cells in the annual growth rings in cross section. Average absolute dry density measurements performed on debarked stem samples of Tora and Björn showed similar values of ca. 468 kg/m^3^ and ca. 473 kg/m^3,^ respectively (Tables 2, 3). Density decreased from ca. 522 to ca. 450 kg/m^3^ in Tora and between ca. 521 and ca. 451 kg/m^3^ in Björn with increasing height from 40 to 400 cm and 40 to 360 cm, respectively (Tables 2, 3). Density measurements at the lower stem heights are consistent with published reports for *S. viminalis* [29] and other *Salix* species [30, 31], while the decrease in density with increasing height is normal for tree growth. Results, however, emphasize the variation that exists and importance of knowing the sample point along the stem.

Since density strongly reflects the anatomical composition, density measurements were also performed on enriched TW regions cut from stems of Tora and Björn from both first- and second growth rings (Table 4). Results for both *Salix* varieties showed a similar trend as the total stem with a decreasing total density between ca. 492–454 kg/m^3^ for Tora and ca. 491–451 kg/m^3^ for Björn with increase in stem height between 80 and 360 cm (Table 4). Density values for the first and second annual rings between 80, 200, 360 cm varied between *Salix* varieties with ca. 424 and 490 kg/m^3^ respectively, recorded for Tora and ca. 451 and 509 kg/m^3^ obtained for Björn for 1^st^ respectively, 2^nd^ growth rings (Table 4). The secondary growth rings were both wider and composed of TW fibres with thicker G-layers. These variations emphasize the inherent differences that are equalized when the density of entire stems are measured. Light- and electron microscopy confirmed variations in the thickness of gelatinous layers especially between early- and latewood (Fig. 6b, f, j).

**Table 4:** Estimated density of tension wood in first and second growth rings of *Salix* varieties

Stem (cm)	Density (kg/m^3^)		Entire sample
	Tension 1st growth ring	Tension 2nd growth ring	
Tora			
360	417.2	488.0	454.4
200	412.1	489.3	462.3
80	443.1	492.6	491.7
Average	424.1	489.9	469.4
Björn			
360	439.6	508.3	451.3
200	457.3	507.5	455.8
80	455.8	512.9	490.9
Average	450.9	509.5	466.0

### G-layer structure, presence of lignin and suitability for enzyme hydrolysis

**Fig. 4:**
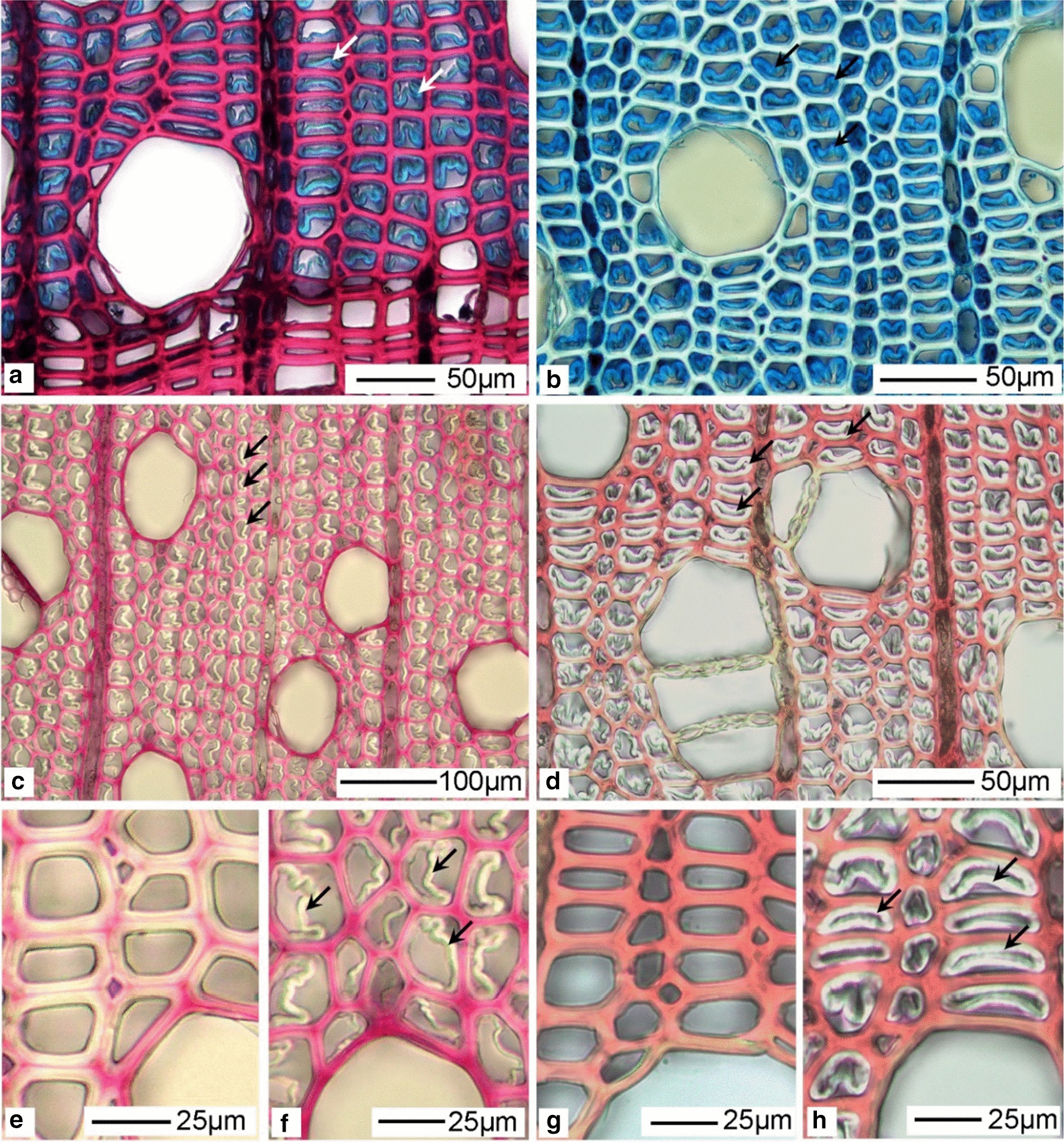
Typical *Salix* Tora TW G-fibres and presence of G-layer (arrows). **a** Safranin/astra blue (double staining) and **b** astra blue only. The G-layer is shown as a thick blue staining layer lining the cell lumina and has been pulled away (i.e., sectioning artefact) in most fibres from the outer fibre secondary cell wall. **c** After treatment with Wiesner (i.e., for distribution of cinnamaldehyde groups), and **d** Mäule (for syringyl-lignin units) reagents, the gelatinous layer reacted negatively and remained almost transparent (arrows). **e**, **f** Mature control (non-TW) and TW, respectively. Red staining indicative of cinnamaldehyde groups is concentrated to the middle lamellae and vessel walls. **g**, **h** Staining of control- (non-TW) and TW with Mäule reagent showing strong staining of fibre secondary walls, a weak reaction with vessel secondary cell wall layers (S1, S2) and negative for the G-layer

Using the sliding microtome approach for sectioning, the G-layer was most often detached from the inner secondary cell wall in studied TW fibre cross sections (Figs. 4a–d, 6). This is typically an artefact of sectioning [16, 32] caused by the weak attachment of the G-layer to the outer secondary wall, likely caused through differences in microfibril angle (MFA) at the interface between the two layers (i.e., G-layer was almost perpendicular to the fibre axis) and the S2 wall (ca. 80–90°) and its lack of lignification (see below). For the purpose of the present study, however, this has less importance as all stem sections were cut in a similar way and any artefacts derived would have been similar for all cross sections and *Salix* varieties. In addition, it is assumed that sectioning would have allowed for improved enzyme penetration and subsequent hydrolysis from both the inside (i.e., interface of the outer secondary wall) and outside (i.e., lumen side) of the G-layer during treatments. SEM observations of control sections further confirmed detachment of the G-layer from the outer lignified secondary cell walls during sectioning (Fig. 6). Previous studies on Poplar, a closely related wood species have also shown differences in MFA, the mesoporous and cellulose aggregate structure of the G-layer as well as its strong affinity for cellulase binding domains [16]. Both the light microscopy and SEM observations confirmed a typical G-fibre organization with a secondary wall structure of S1 + S2 + G layers as previously reported for *Salix* varieties [33]. While the G-layer varied considerably in thickness between growth rings and both early- and latewood, the G-layer appeared as an “open” rather than compact structure as previously reported for other wood species with similar S1 + S2 + G cell wall structure [16, 26].

Light microscopy of TW fibres typically showed strong staining of the G-layer with astra blue in single and double staining approaches consistent with lack of lignin [34] and open structure (Fig. 4a, b). Lack of lignin in the G-layer was confirmed by negative reactions with both Weisner and Mäule reagents (Fig. 4c–h), the former staining middle lamellae and vessel secondary walls strongly reddish-pink for cinnamaldehyde units (i.e., guaiacyl lignin) and the latter the outer secondary walls in G-fibres and control normal fibres for syringyl lignin units (Fig. 4e–h). Previous studies have further confirmed the presence of a variety on non-cellulosic components, particularly 1-4-β-d galactan in the G-layer of *Salix* spp. [33]. Both the open structure and lack of lignification of the G-layer together with cross sections and open fibres enhanced the possibility of both enzyme penetration and hydrolysis.

### Enzymatic hydrolysis of G-layer cellulose in *Salix* stem cross sections-morphological observations

**Fig. 5:**
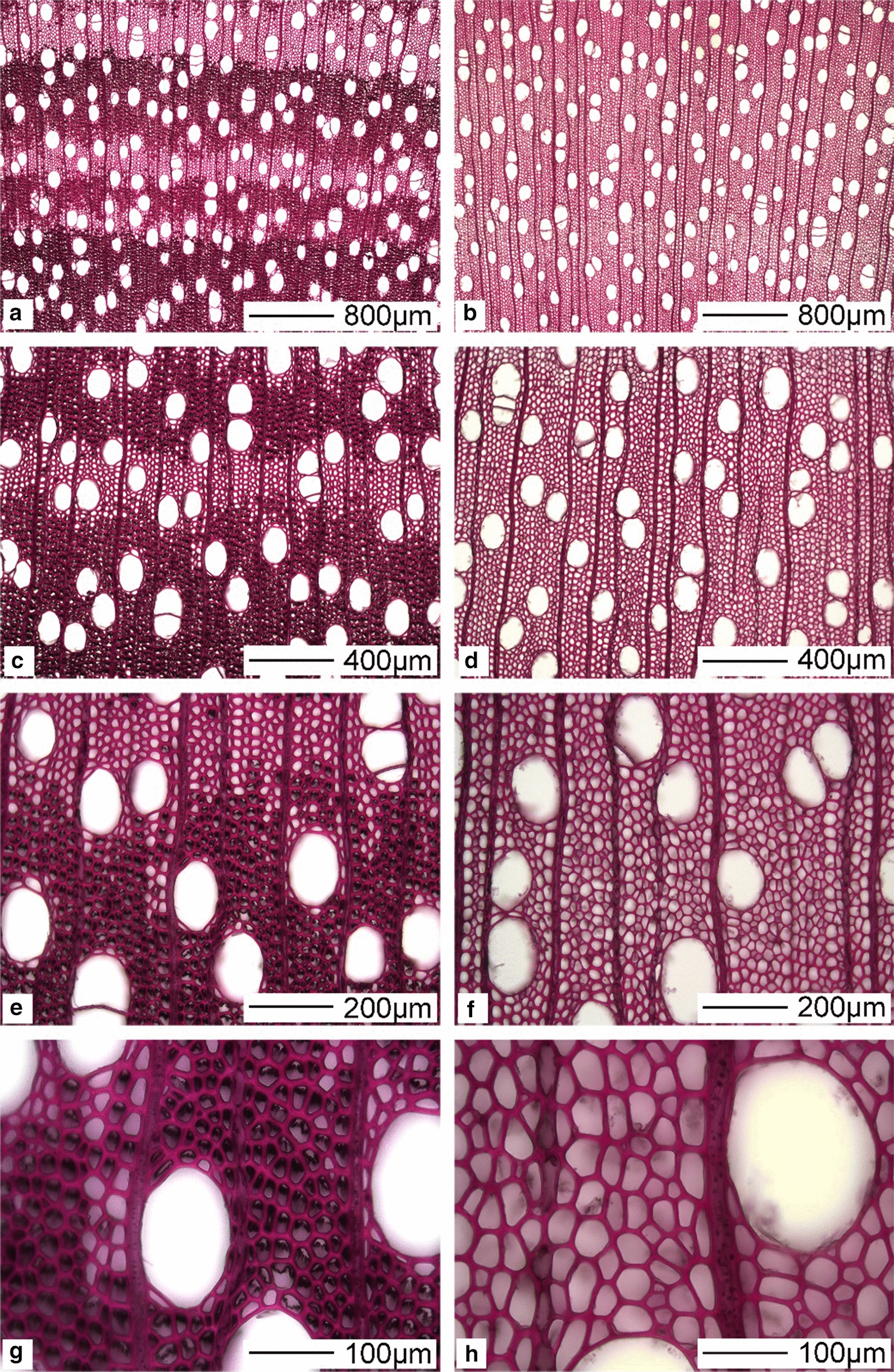
Light microscopy of Tora TW G-fibres before (left column; **a**, **c**, **e** and **g**) and after (right column; **b**, **d**, **f**, and **h**) cellulase treatment and double staining with chlorazol black E and safranin. In controls, the TW shows strongly staining bands of G-fibres (**a**, **c**, **e**, **g**) who’s G-layer was hydrolyzed after cellulase treatment (right column)

**Fig. 6:**
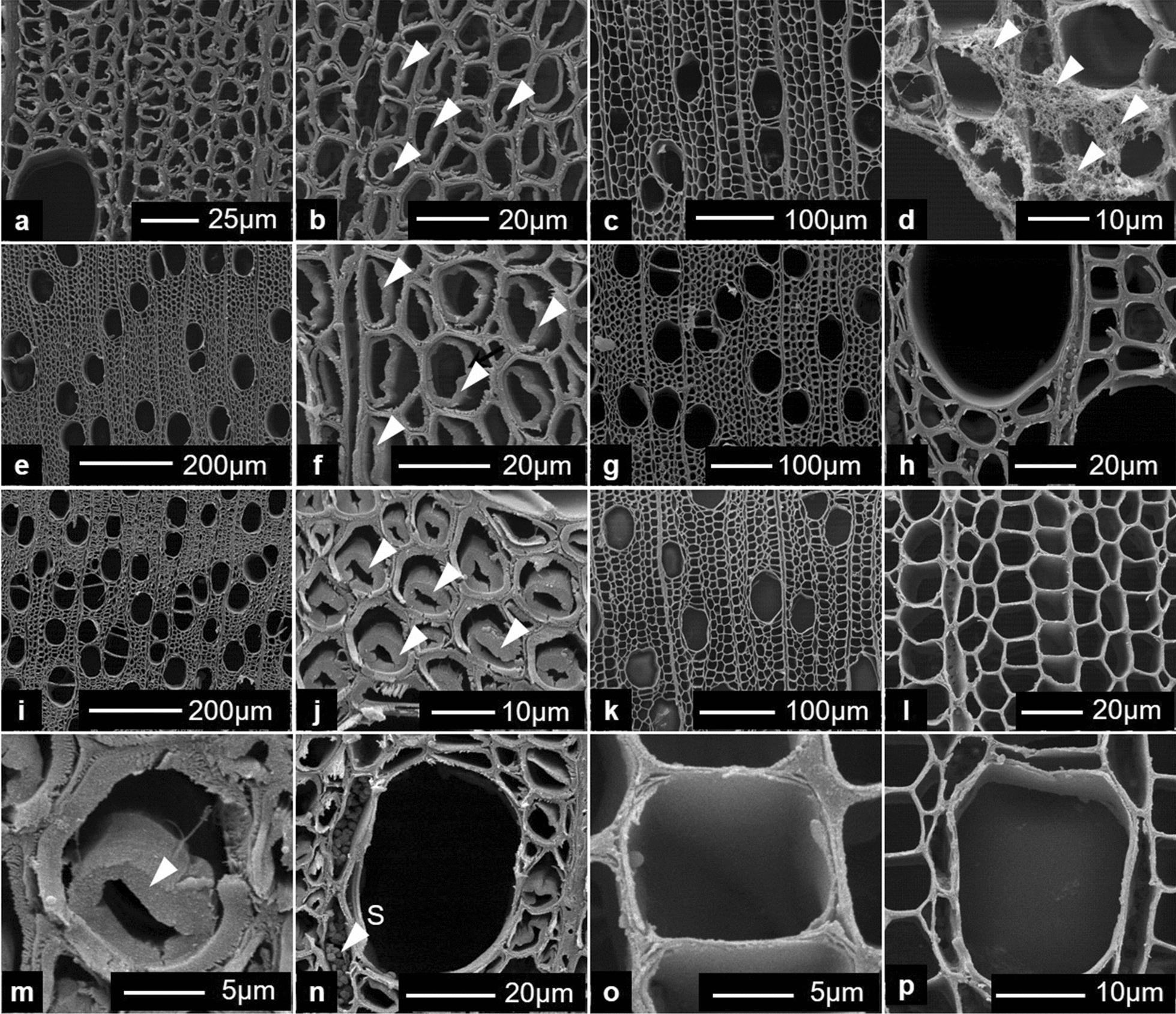
Scanning electron micrographs of TW fibres and G-layers from *Salix* Tora stem cross sections. Control (**a**, **b**, **e**, **f**, **i**, **j**, **m**, **n**) and after 2- (**d**) and 3-day treatment with cellulase (Cellic CTec2) (**c**, **g**, **h**, **k**, **l**, **o**, **p**). Top row (**a**–**d**) from 40 cm height of the stem, row 2 (**e**–**h**) from 200 cm and rows 3 and 4 (**i**–**p**) from 360 cm height. The G-layer varied in thickness between different growth rings and between early- and latewood being recognized as an extra layer lining the secondary cell wall (arrowheads, **b**, **f**, **j**, **m**). The G-layer was hydrolyzed after 3-day cellulase treatment (**c**, **g**, **h**, **k**, **l**, **o**, **p**). Morphological changes of the secondary cell walls and the compound middle lamellae between fibres, vessels and parenchyma cells and storage materials [e.g., starch granules, **n**. (S)] through enzyme treatment were not apparent. Occasional fibrous remnants of the G-layer were observed on sections treated for shorter periods (**d**, arrowheads). Enzyme treatment caused removal of the G-layers

Initial studies were performed to determine optimal conditions for using the cellulase complement Cellic CTec2 on *Salix* stem cross sections. Using the recommended enzyme dosage (i.e., enzyme concentration 20µL/10 mL citrate buffer), temperature and pH [35] and 80–100 mg biomass dry weight, we found by monitoring d-glucose release and progressive G-layer removal using light microscopy observations, that 3 days was optimal (see “Materials and methods”). We also found that pre-drying cross sections at 103 ± 2 °C for dry wt determinations before enzyme treatment did not reduce d-glucose production. Shorter periods of enzyme incubation resulted in partially degraded G-layers, but indicated enzyme hydrolysis and surface erosion from directions of both the fibre lumen and interface between the outer fibre secondary cell walls and the G-layer. Occasional fibrous material of partially hydrolyzed G-layers was also found on the sections incubated for shorter periods (Fig. 6d). Light microscopy of stained sections showed almost complete removal of TW bands and G-layers after 3-day incubation leaving only a very thin residue layer in same fibres (Fig. 5a–h). Similarly, SEM observations after 3-day enzyme treatment of cross sections from stems at 40, 200 and 360 cm height showed the absence of G-layers (Fig. 6c, d, g, h, k, l, o, p). Light- and SEM microscopy was used to observe the lignified wood cells for evidence of cell wall hydrolysis (e.g., cavity formation, surface erosion). No morphological evidence for hydrolysis and attack of lignified cell walls in fibres and vessel secondary cell walls and middle lamellae was found using this approach suggesting that enzyme digestion after 3 days was primarily directed at the non-lignified cellulose-rich gelatinous layers in the cross sections. Additional observations were made on 1-day enzyme-treated radial- and tangential longitudinal sections cut from *Salix* stems with TW and G-fibres. This showed similar evidence for the surface hydrolysis of G-layers (not shown). Storage materials such as starch granules also remained in vasicentric parenchyma cells surrounding vessels after enzyme treatment (Fig. 6n). Observations indicate the G-layers were readily accessible to hydrolysis by the cellulase and that digestion was primarily from both sides of the G-layers in sections.

The lack of morphological evidence for attack of the cell walls of other cellular elements (i.e., vessels, parenchyma cells, outer fibre secondary cell walls) provides further evidence for the importance of biomass pre-comminution and need for the exposure of cellulose during cellulase hydrolysis of lignocellulose. Despite using cross sections, little attack was apparent of the fibre cut surfaces, suggesting that the enzyme complement used had greater endo-cellulase than exo-cellulase activity on the *Salix* samples.

### Quantitation of accessible d-glucose in *Salix* stems using Cellic CTec2 and GOPOX assay

**Fig. 7:**
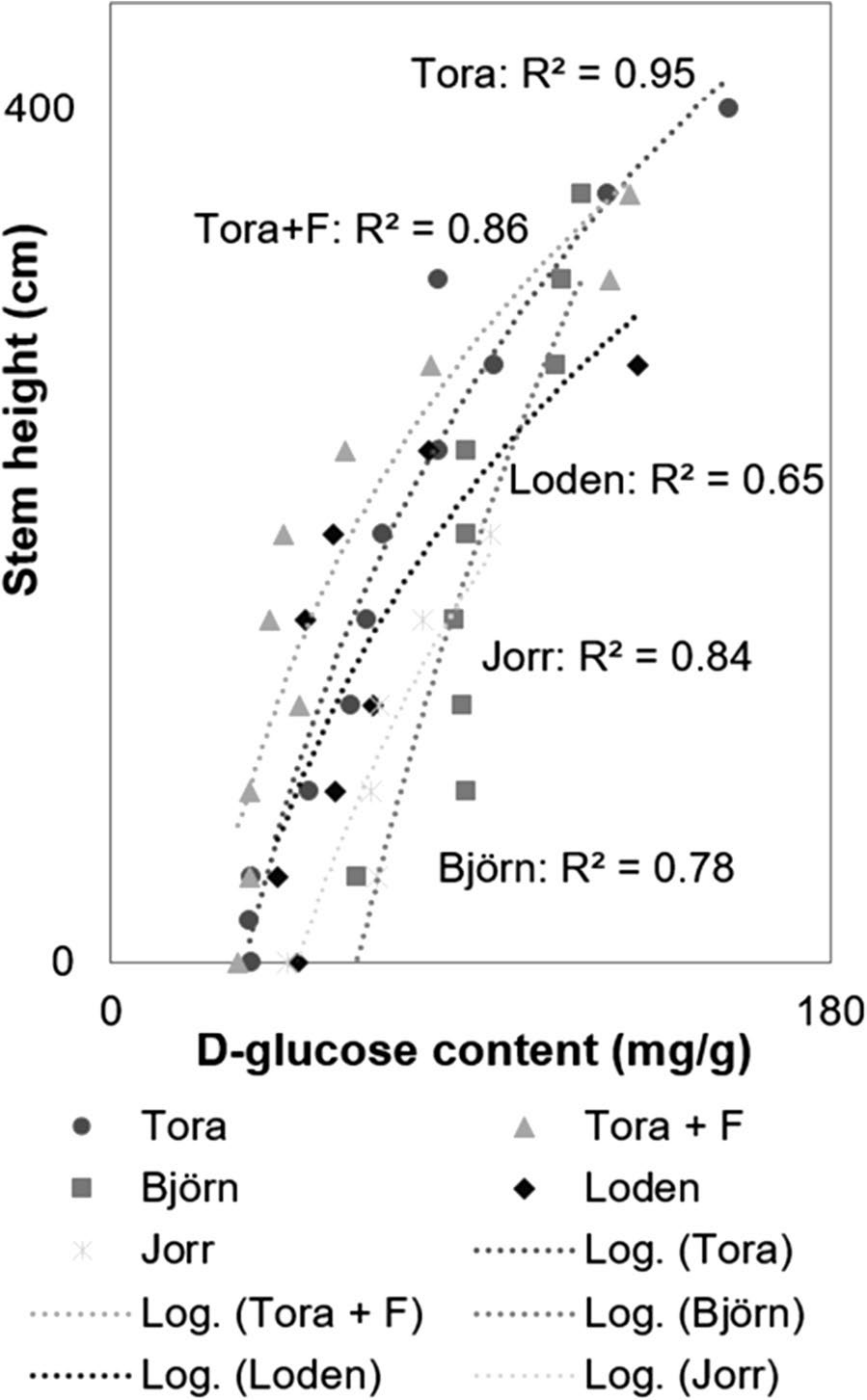
d-Glucose content (mg/g dry wt *Salix*) as measured along stems of variants: Tora, Tora + fertilizer (Tora + F), Björn, Loden and Jorr

**Fig. 8:**
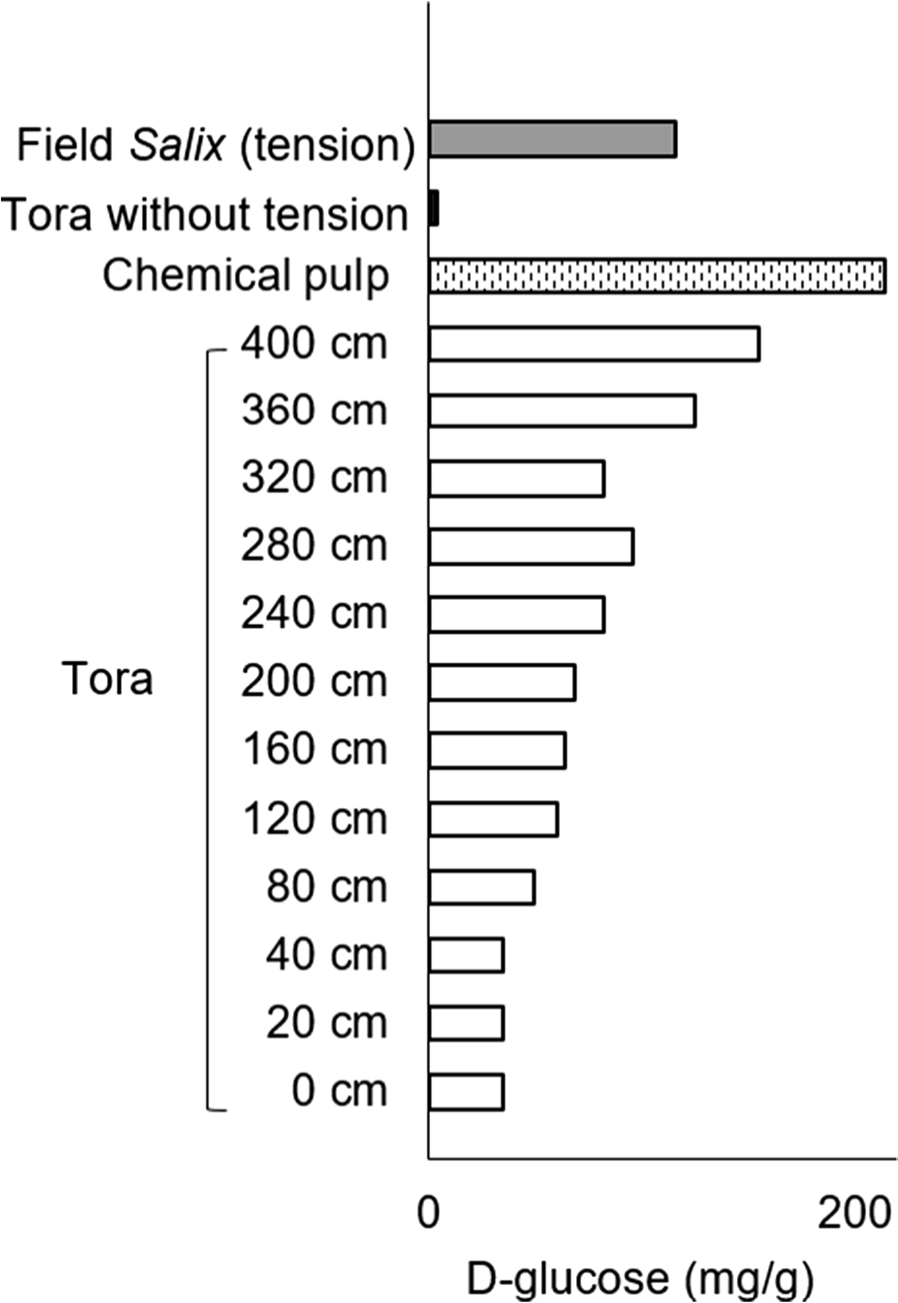
Distribution of measured d-glucose in mg/g dry wt of Tora stem and controls: (1) bleached chemical softwood pulp; (2) Cellulase pretreated Tora sections (i.e., without TW); and (3) TW cross sections from a field 8-year-old *Salix* sample

**Fig. 9:**
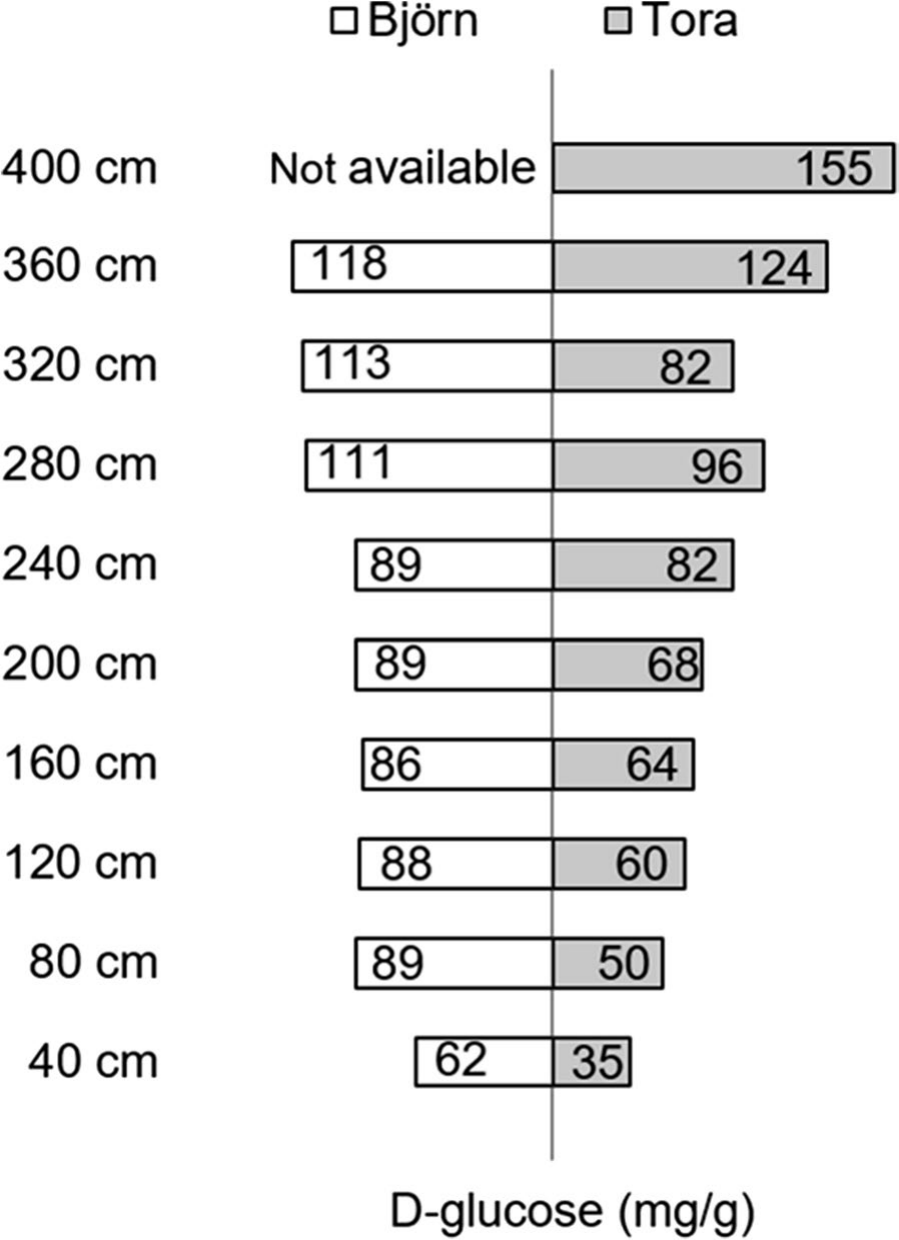
Comparison between distribution of measured d-glucose in mg/g dry wt along the stems of *Salix* varieties Björn and Tora

Figure 7 shows quantification of the progressive release of d-glucose after Cellic CTec2 treatment over 3 days for 4 *Salix* varieties and one variety after fertilization (Tora + F). Values of R^2^ ranged from 0.78 (lowest) for Björn and 0.95 for Tora showing increased d-glucose production with *Salix* stem height for all varieties (Fig. 7). Additional R^2^ values included 0.64 for Loden, 0.84 for Jorr and 0.86 for Tora + F (Fig. 7). Results show a good correlation providing evidence for a progressive and stepwise release of d-glucose and absence of inhibition. Figures 8, 9 and Tables 2, 3 show results for the final release of d-glucose following 3-day cellulase treatment of stem cross sections from 40 to 400 cm for Tora and 40 to 360 cm for Björn. Results for both *Salix* varieties show an increase of d-glucose release with stem height with final values of ca. 156 (Tora) and ca. 118 (Björn) mg of d-glucose /g dry wt of *Salix* wood, respectively. Figure 8 further shows the release of d-glucose from material controls including a bleached spruce pulp (d-glucose 214 mg/g dry wt), pre-hydrolyzed (i.e., pre-cellulase treated) cross sections of Tora (d-glucose 4 mg/g dry wt) and an 8-year field *Salix* sample (d-glucose 116 mg/g dry wt). The contrasting d-glucose production with the bleached softwood pulp and pre-hydrolyzed *Salix* cross sections is consistent with lack of lignin and freely available cellulose in the former and lack of freely available cellulose through lignification in the latter. Results with the field *Salix* TW sample are also consistent with freely available G-layer cellulose and the analysis method, although it is not known if age can change cellulose availability.

**Fig. 10:**
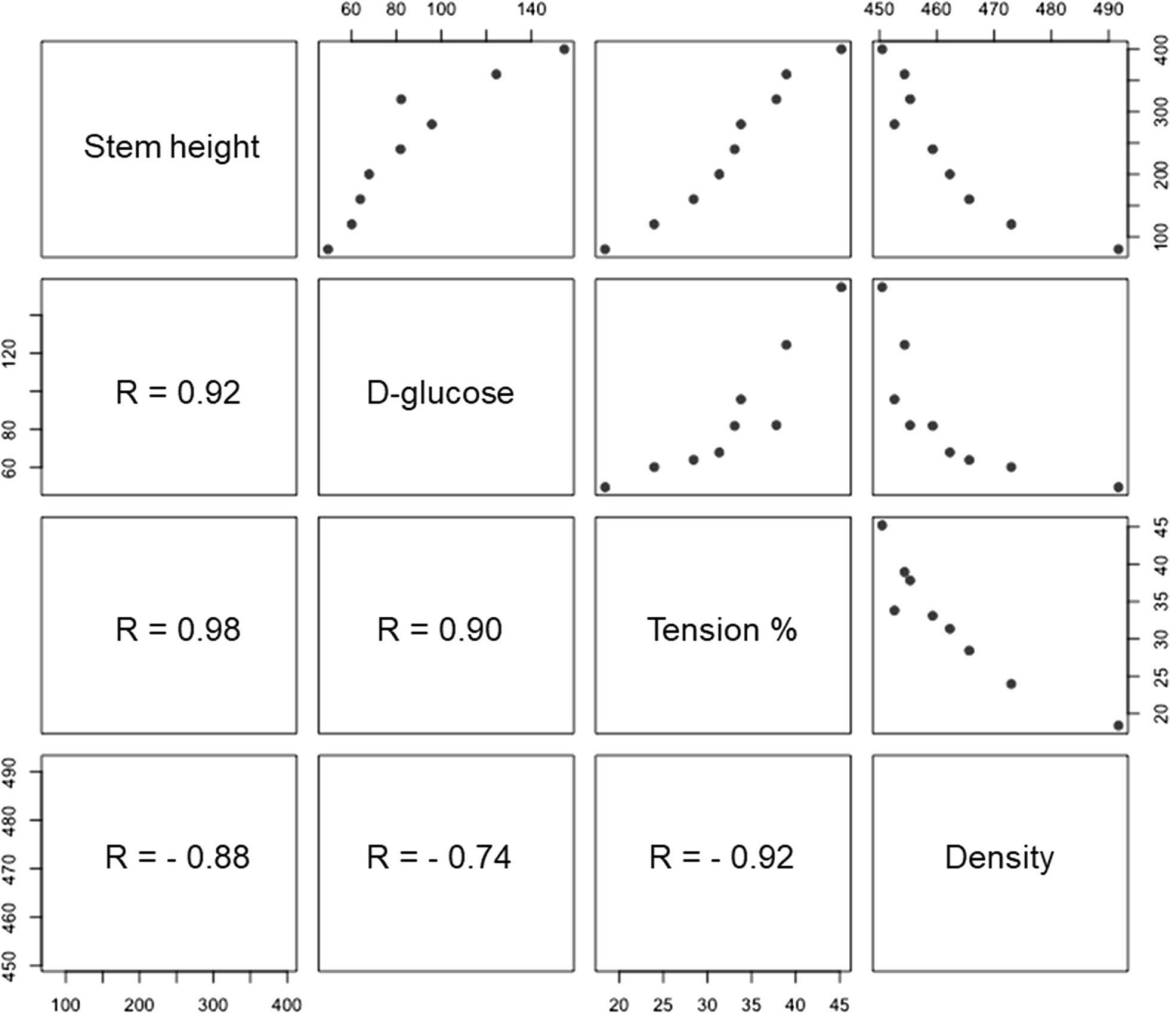
Scatterplot matrix showing correlations between stem height (cm), d-glucose (mg/g dry wt), percentage TW in stem cross sections (%) and density (kg/m^3^) for Tora. The strength of the relationship is given by the correlation coefficient value R. Positive correlations were given between stem height and d-glucose and percentage TW and negative correlations between stem densities (total), stem height, d-glucose and percentage TW

Results indicate variations in available G-layer along the stem and presumably reflect cellulose accessibility. The value of d-glucose release (i.e., ca. 156 mg/g dry wt) for Tora at 400 cm approaches that for the bleached softwood pulp, where accessibility for cellulose should not be impeded. Minimal d-glucose release for the pre-hydrolyzed cross sections similarly reflect the lack of freely available cellulose, while the value for the 8-year *Salix* field sample indicates the method will also be viable for older stem material (Fig. 8). Comparison of the final release of d-glucose from Tora and Björn shows the latter *Salix* varieties to release greater amounts between 40 and 320 cm and in the latter higher release at 360 cm and above (Fig. 9, Tables 2, 3). Additional analysis of d-glucose release from *Salix* varieties Tora plus fertilization, Loden and Jorr with stem diameters of 1.62–1.72 (Table 5) showed stems with larger diameter to release more d-glucose (e.g., Björn). d-glucose release at height 200 cm showed variations between the different varieties (Table 6), although this was probably more likely related to the final height attained as the upper regions always gave highest release of d-glucose and the *Salix* varieties showed differences. Figure 10 shows a scatterplot matrix of the four variables, stem height, d-glucose production, percentage TW and density. Strong positive correlations were given between stem height, d-glucose and percentage TW all of which correlated negatively with density.

**Table 5:** Estimation of d-glucose for five *Salix* varieties from stem sections with diameter 1.62–1.72 cm

Stem diameter between 1.62 and 1.72 cm		
	d-Glucose content (mg/g dry wt)	Stem height (cm)
Tora	96	280
Tora + F	80	280
Björn	113	320
Loden	56	80
Jorr	65	80

**Table 6:** Estimation of d-glucose for five *Salix* varieties from sections taken at a height of ~ 200 cm

At the height ~ 200 cm		
	d-Glucose content (mg/g dry wt)	Stem diameter (cm, without bark)
Tora	68	2.04
Tora + F	43	1.98
Björn	89	2.06
Loden	95	1.20
Jorr	56	1.36

### Estimation of total d-glucose release from stems of different *Salix* varieties and relation with total cellulose

For bioethanol production, one of the most important criteria is the total native cellulose present in the stem biomass and its accessibility for first stage enzyme (cellulase) hydrolysis [1]. With cellulose accessibility, this is accepted as regulated by biomass recalcitrance, where both lignin type and concentration can play an important role [36]. Previous studies on *Salix* varieties using wet chemical analysis have reported total cellulose contents ranging between 41.6 and 55.9% (i.e., mean 45%) with corresponding lignin levels between 13.8 and 28.0% [2, 37–42]. Therefore, for our calculations on the possible contribution played by G-layer cellulose to total stem cellulose concentration in our samples, we used 45% cellulose. From our staining experiments with stem cross sections, the gelatinous layer appeared non-lignified and thus the cellulose should be freely available for enzymatic hydrolysis, and, therefore, should not be affected by recalcitrance. Using these criteria and a total stem volume of 40–400 cm, the total calculated free d-glucose available that could be produced through the enzymatic treatment of the G-layer in TW in the Tora stems analyzed would lie in the region ca. 16% of the total cellulose (Table 7). If the total stem growth between 0 and 400 cm is used (i.e., not considering the primary shoot growth above), then the calculated value is ca. 14%. The corresponding values for Björn between 80 and 320 cm (% TW were not calculated at 40 and 360 cm; i.e., only the 1st annual ring was present in the latter) were ca. 21% and 20%, respectively (Table 8). Additional analysis of the 8-year-old *Salix* field sample (i.e., contained only TW) taken as control and at only one point in the stem was ca. 26% (Table 7). These results indicate that between ca. 15 and 21% of the total cellulose (i.e., 45%) in Tora and Björn stems maybe contributed by accessible cellulose in the gelatinous layers of TW fibres. Brereton et al. [43], using a 3-month-old laboratory cultivated *Salix* variant with tension induction, found that isolated pure TW after milling to fine particles could release ca. 250 mg/g dry wt glucose with enzymatic saccharification, which was 66% higher than the corresponding control (released ca. 151 mg/g dry wt glucose). For the genotype Tora, which is the same *Salix* variety used in our study, the glucose yield was 150 mg/g dry wt after 3 years growing under low reaction wood inducing conditions, and 200 mg/g dry wt after 4 years growing under high reaction wood inducing conditions [2]. Sassner [3] also recorded a very high enzymatic glucose yield from 4-year-old Tora that released 55.6 g glucose/100 g dry wt after steam pretreatment, sulphuric acid impregnation and subsequent enzymatic hydrolysis.

**Table 7:** Total d-glucose as a percentage of total cellulose for Tora

Tora	Stem height (cm)	Stem weight (g)	Stem volume (cm^3^)	Tension volume (cm^3^)	d-Glucose amount (g)
Every 40 cm	400–360	20.7	45.9	20.8	3.2
	360–320	29.7	65.4	25.5	3.7
	320–280	37.2	81.6	30.8	3.1
	280–240	46.2	102.1	34.5	4.4
	240–200	56.3	122.6	40.6	4.6
	200–160	65.3	141.3	44.2	4.4
	160–120	75.8	162.7	46.2	4.8
	120–80	88.2	186.4	44.7	5.3
	80–40	110.8	225.4	41.5	5.5
	40–0	131.4	251.5		4.6
Entire stem	400–0	661.6	1385.0		43.7
Percentage of tension volume (40–400 cm): 29.0%					
Total d-glucose/total cellulose (0–400 cm): 14.7%					
Total d-glucose/total cellulose (40–400 cm): 16.4%					
Field *Salix* sample’s total d-glucose/total cellulose: 25.78%

**Table 8:** Total d-glucose as a percentage of total cellulose for Björn

Björn	Stem height (cm)	Stem weight (g)	Stem volume (cm^3^)	Tension volume (cm^3^)	d-Glucose amount (g)
Every 40 cm	360–320	29.7	70.2		3.7
	320–280	37.2	92.1	35.1	4.9
	280–240	46.2	108.1	40.7	5.6
	240–200	56.3	123.9	40.8	5.1
	200–160	65.3	142.6	46.6	5.8
	160–120	75.8	162.7	52.9	6.5
	120–80	88.2	187.2	52.6	7.8
	80–40	110.8	217.5	57.6	9.5
	40–0	131.4	234.0		7.5
Entire stem	360–0	640.9	1338.4		56.5
Percentage of tension volume (80–320 cm): 31.6%					
Total d-glucose/total cellulose (0–360 cm): 19.6%					
Total d-glucose/total cellulose (80–320 cm): 20.6%					

TW is normally recognized as an abnormal wood trait developed by many angiosperm trees that allows them to maintain its branches in a perpendicular position to the main trunk. TW development in branches is thought to be induced as a gravitational response [13], while the mechanism in young, rapidly growing coppice trees like *Salix* is unknown. Studies have shown, however, that both genotype and phenotypic response can be important for coppice trees, with one study showing a fivefold increase in glucose release from a *Salix* variety grown under control- and adverse windy conditions [15]. Quantitative analysis of percentage TW in stems in the present study grown at the same site showed variations with stem height and between annual growth rings. Similarly, it was difficult to separate the microdistribution of TW into classical zones of TW, opposite wood and normal wood as TW was frequently developed multilaterally around stems.

The present study further emphasizes the important contribution that TW and G-layer cellulose may have on the total cellulose content of *Salix* grown under field situations, a result consistent with previous studies and important with respect to optimizing biofuel production from coppice trees [15, 17, 44]. The use of stem cross sections provides a novel approach, whereby discrete stem regions and entire stems can be assessed directly for quantifying the total TW G-cellulose content in stems, thereby providing information on its accessibility as a readily available source of hydrolysable cellulose. Observations further confirmed the important role played by lignin for recalcitrance in the secondary cell walls of both the outer cell wall layers in G-fibres and other cell elements that remained undigested during cellulase treatments in comparison to the non-lignified gelatinous layer of TW fibres. This result further confirms the importance of biomass comminution and cellulose exposure for cellulases during biomass processing. Results, therefore, indicate a very important contribution can be played by presence of TW and G-layer cellulose and that this contribution likely varies with genotype and prevailing environmental conditions.

## Conclusions

Physical, histochemical/microscopy, image analysis and enzymatic studies were performed on 2-year-old rapidly growing *Salix* varieties to quantify the presence of tension wood (TW) along stems, characterize TW G-fibres and determine the likely contribution played by gelatinous layer cellulose to total stem cellulose;

A method involving exposure of cross sections cut from the stems of different *Salix* varieties was developed and allowed determination of the role played by the G-layer cellulose from TW fibres;

Density, microscopy (LM/SEM) and image analysis methods confirmed multilateral TW development in all stems and varieties with increasing % TW negatively correlated with density but positively correlated with stem height and cellulose (as d-glucose);

G-layer cellulose in TW fibres exposed in cross sections was readily hydrolyzed by cellulase producing ca. 44 and 38 kg/m^3^ of d-glucose on average for entire stems of *Salix* varieties Björn and Tora, respectively, with the d-glucose release increasing with stem height;

By correlating stem TW volume with average total cellulose content from previous chemical analysis (i.e., literature value. 45%), it was estimated that G-layer cellulose contributed between 16 and 20% of the total cellulose yield in the two *Salix* varieties (Tora, Björn) studied.

## Materials and methods

### Plant materials and harvesting

The 2-year-old *Salix* stems used were derived from a short rotation plantation experiment on arable land in Uppsala, Central Sweden (59° 49′ N, 17° 39′ E) [11], September 2020. The *Salix* varieties were taxonomically distinct at species or genotype level and included two full siblings ‘Björn’ (*Salix schwerinii* E. Wolf. × *S. viminalis* L.) and ‘Tora’ (*S. schwerinii* × *S. viminalis*) as well as ‘Jorr’ (*S. viminalis*) and ‘Loden’ (*S. dasyclados* Wimm.), the latter most distinct in terms of taxonomy from the other three varieties [11]. The stems were selected from a randomized plot, cut ca. 10 cm above the soil surface, transported to the laboratory, and maintained at -20° C until further use. Physical features (stem length and breadth) were recorded in the laboratory before further analyses. Stems were sawn at 10 cm intervals from the basal end of the stem (Fig. 2).

### Density measurements

Absolute dry densities of wood along *Salix* stems were calculated for debarked cylinders of ca. 2 cm sawn every 40 cm from the basal end of the stem (Fig. 1, Tables 2, 3). The 2 cm wood cylinders were dried in an oven at 103 ± 2 °C overnight and absolute dry weight measured next day. The volume of individual wood samples was then measured by the water displacement method [45]. Wood density (*ρ*) was determined as the mass (i.e., dry wt.) divided by volume and given in kg/m^3^. To determine the effect of TW on wood density, areas rich in TW (ca. 0.3–5 mm in cross section × 2.0 cm long), from Tora and Björn at 80, 200, 360 cm above ground, were carefully cut from the primary- and secondary growth rings of stems (Table 4) and processed as described above. Serial cut samples (2–3) were used for each measurement and each sample was measured three times.

### Sectioning and staining of stem cross sections for tension wood

Entire stem cross sections (ca. 15–30 µm thick, Fig. 1) were cut using a sledge sliding microtome (Leica 1300 sledge microtome). Serial sections were stained initially with 1% w/v astra blue or double stained with either 1% w/v astra blue + 0.1% w/v safranin, 1% w/v chlorazol black E + 0.1% w/v safranin) [46] to visualize presence of TW. Chlorazol black E and astra blue stain the gelatinous layer (G-layer) of TW fibres strongly black or blue. The combination of safranin and chorazol black E gave best staining (Fig. 1) for subsequent image analysis. Entire stem cross sections were stained and scanned using an Epson Perfection Pro 750 film scanner with a pixel resolution of 2400 dpi and areas of TW marked. Thereafter, using Adobe Photoshop CC 2017, the pixel values of the selected TW area and the whole section area can be determined. The percentage area of TW in entire stem cross sections was then quantified using the equation: area of TW in pixels / area of the whole section in pixels × 100%. From this, the total volume in kg/m^3^ for individual sections of the stem could be calculated. Detailed analyses of individual TW regions and fibres after staining was conducted using a Leica DMLB light microscope (Leica Microsystems, Wetzlar, Germany) equipped with an Infinity X-32 digital camera (Deltapix, Samourn, Denmark) to confirm presence/absence of TW and G-layers. To visualize lignin distributions, fresh sections were stained using the Wiesner and Mäule reactions [47, 48]. For the Wiesner reaction, sections were treated with 2% v/v phloroglucinol in ethanol and mounted in 6 M hydrochloric acid. For the Mäule reaction, sections were stained with 1% w/v potassium permanganate for 5 min, washed (5 min in water), treated with 2 M HCl for 5 min, washed (5 min in water) and mounted in ammonium hydroxide. Sections were observed directly after Wiesner and Mäule reactions, as the colour reaction is unstable. All stains were purchased from Sigma-Aldrich (St. Louis, USA).

Serial semi-thin cross sections of stems (ca. 15–30 µm thick, Fig. 1) were also cut for the staining and enzyme treatments using a commercial cellulase complement (Cellic CTec2, Novozymes [35]) and the light- and electron microscopy studies described below. In addition, radial- and transverse longitudinal sections (ca. 15–30 µm thick) cut from *Salix* stem regions showing TW were treated with cellulase to visualize changes in the morphology of the G-layer during enzymatic hydrolysis.

### Enzyme hydrolysis

All studies were conducted on debarked and washed (distilled water) serial stem sections. Initial studies were carried out on stem sections of Tora to determine optimal conditions for incubation with Cellic CTec2, a cellulase blend enzyme for producing d-glucose [35]. According to the manufacturers, the enzyme blend contains a cocktail of cellulases and recommended for industrial treatment of lignocellulose biomass [35]. This involved time-period, enzyme concentration, number of *Salix* sections sufficient for quantification of d-glucose production, and studies on the effect of pre-drying sections at 103 ± 2 °C for dry weight determination before enzyme treatment as well as observations on the progressive removal of the G-layer using light microscopy after staining with chlorazol black E and safranin. Detection of free d-glucose was done using the glucose oxidase/peroxidase (GOPOD) assay kit according to the manufacturer’s instructions (Megazyme, Bray, Ireland). Stem cross sections were incubated in 50 mL Erlenmeyer flasks containing 20 µL Cellic CTec2 enzyme in 10 mL citrate buffer (pH 5), at 50 °C and 100 rpm (i.e., sufficient to keep sections slightly moving and separate from each other) for 3 days in an INNOVA 4000 rotary incubator (Instrument AB, Lambda, Sweden). Twenty microliters of 95% ethanol was used as bactericide. A minimum of three serial stem cross sections (i.e., ca. 80–100 mg wood, dry wt) were used starting at 40 cm above the ground from *Salix* varieties (Tables 2, 3, 5, 6; Figs. 2, 3). Additional enzymatic analyses were conducted on an 8-year field sample of *S. viminalis* (Fig. 8).

As positive control for TW, washed stem sections of Tora previously incubated with Cellic CTec2 and shown by light microscopy to have their G-layers in TW fibres hydrolyzed were used. An obvious control would have been opposite or mature wood from similar stem regions. However, as shown from the total distribution and percentage of TW in the *Salix* stems (Figs. 2, 3), separating such samples was difficult. An additional method control included bleached (softwood) chemical pulp fibres [49]. Results of enzyme activity are expressed as mg d-glucose produced per g dry wt of wood material from sections or in kg/m^3^ when whole stems are compared. All experiments were made in duplicate, reflecting a minimum of three serial cross sections per flask depending on stem cross-sectional area. For enzymatic analysis of sections from the upper regions of stems, where the diameter of the stem was small (Tables 2, 3), a minimum of 5–10 sections was required for quantification of d-glucose.

### Scanning electron microscopy

Scanning electron microscopy (SEM) was used to follow the progressive hydrolysis of TW G-layers as well as any hydrolytic effects of the cellulase complement on the cellular structure of other cell wall layers (i.e., secondary cell walls/primary, middle lamella regions). Entire semi-thin sections of stems after enzymatic treatment and controls were fixed in 3% v/v glutaraldehyde in 0.1 M sodium cacodylate buffer (pH 7.2) for 3 h at room temperature. After washing in the same buffer (3 × 20 min), sections were post fixed in 1% w/v osmium tetroxide for 1 h. Following washing in water (3 × 30 min; and overnight), sections were dehydrated in a progressive series of ethanol [16] and finally acetone. Samples were dried in an Agar E3000 critical point dryer (Quorum Technologies Ltd., East Essex, UK) using liquid CO_2_ as the drying agent, mounted on stubs, and coated with gold using an Emitech K550X sputter device (Emitech Ltd., Kent, UK) [16]. Observations were made using a Philips XL 30 ESEM (Philips, Eindhoven, Netherlands) operated at 10–20 kV with images recorded digitally.

### Analysis of tension wood in stems from field experiment

For comparison, a stem from an 8-year-old growing tree of *S. viminalis* was cut to provide control wood materials for the enzymatic studies and calculations of d-glucose production.

## Data Availability

The data and material used to support the findings of this study are included within the article.

## References

[1] Ledin S (1996). Willow wood properties, production and economy. Biomass Bioenergy.

[2] Sassner P, Galbe M, Zacchi G (2008). Techno-economic evaluation of bioethanol production from three different lignocellulosic materials. Biomass Bioenergy.

[3] Serapiglia MJ, Cameron KD, Stipanovic AJ, Abrahamson LP, Volk TA, Smart LB (2013). Yield and woody biomass traits of novel shrub willow hybrids at two contrasting sites. Bioenergy Res.

[4] Pawar PMA, Schnürer A, Mellerowicz EJ, Rönnberg-Wästljung AC (2018). QTL mapping of wood FT-IR chemotypes shows promise for improving biofuel potential in short rotation coppice willow (*Salix* spp.). Bioenergy Res..

[5] Estévez V, Villacampa M, Menéndez JC (2014). Recent advances in the synthesis of pyrroles by multicomponent reactions. Chem Soc Rev.

[6] Horn SJ, Estevez MM, Nielsen HK, Linjordet R, Eijsink VG (2011). Biogas production and saccharification of *Salix* pretreated at different steam explosion conditions. Bioresour Technol.

[7] Weih M, Glynn C, Baum C (2019). Willow short-rotation coppice as model system for exploring ecological theory on biodiversity—ecosystem function. Diversity.

[8] Sjostrom E (1993). Wood chemistry: fundamentals and applications.

[9] Foston M, Trajano HL, Samuel R, Wyman CE, He J, Ragauskas AJ (2015). Recalcitrance and structural analysis by water-only flowthrough pretreatment of ^13^C enriched corn stover stem. Bioresour Technol.

[10] Ray MJ, Brereton NJ, Shield I, Karp A, Murphy RJ (2012). Variation in cell wall composition and accessibility in relation to biofuel potential of short rotation coppice willows. Bioenergy Res.

[11] Weih M, Nordh N-E, Manzoni S, Hoeber S (2021). Functional traits of individual varieties as determinants of growth and nitrogen use patterns in mixed stands of willow (*Salix* spp.). For Ecol Manage.

[12] Weih M, Hansson P, Ohlsson J, Sandgren M, Schnürer A, Rönnberg Wästljung A. Sustainable production of willow for biofuel use. Achieving carbon—negative bioenergy systems from plant materials. Edited by Saffron C. Cambridge, UK: Burleigh Dodds Science Publishing Limited; 2020. p. 1–36.

[13] Gardiner B, Barnett J, Saranpää P, Gril J (2014). The biology of reaction wood.

[14] Ruelle J (2014). Morphology, anatomy and ultrastructure of reaction wood. The biology of reaction wood.

[15] Brereton NJ, Ray MJ, Shield I, Martin P, Karp A, Murphy RJ (2012). Reaction wood–a key cause of variation in cell wall recalcitrance in willow. Biotechnol Biofuels.

[16] Daniel G, Filonova L, Kallas ÅM, Teeri TT (2006). Morphological and chemical characterisation of the G-layer in tension wood fibres of* Populus tremula* and* Betula verrucosa*: Labelling with cellulose-binding module CBM1_*Hj*Cel7A_ and fluorescence and FE-SEM microscopy. Holzforschung.

[17] Berthod N, Brereton NJ, Pitre FE, Labrecque M (2015). Five willow varieties cultivated across diverse field environments reveal stem density variation associated with high tension wood abundance. Front Plant Sci.

[18] Dadswell H, Wardrop A (1949). What is reaction wood?. Aust For.

[19] Furuya N, Takahashi S, Miyazaki M (1970). The chemical composition of the gelatinous layer from the tension wood of* Populus euroamericana*. J Jpn Wood Res Soc.

[20] Norberg PH, Meier H (1966). Physical and chemical properties of the gelatinous layer in tension wood fibres of aspen (*Populus tremula* L.). Holzforschung-Int J Biol Chem Phys Technol Wood.

[21] Kaku T, Serada S, Baba KI, Tanaka F, Hayashi T (2009). Proteomic analysis of the G-layer in poplar tension wood. J Wood Sci.

[22] Nishikubo N, Awano T, Banasiak A, Bourquin V, Ibatullin F, Funada R, Brumer H, Teeri TT, Hayashi T, Sundberg B (2007). Xyloglucan endo-transglycosylase (XET) functions in gelatinous layers of tension wood fibers in poplar—a glimpse into the mechanism of the balancing act of trees. Plant Cell Physiol..

[23] Wada M, Okano T, Sugiyama J, Horii F (1995). Characterization of tension and normally lignified wood cellulose in *Populus maximowiczii*. Cellu.

[24] Donaldson L (2007). Cellulose microfibril aggregates and their size variation with cell wall type. Wood Sci Technol.

[25] Clair B, Gril J, Di Renzo F, Yamamoto H, Quignard F (2008). Characterization of a gel in the cell wall to elucidate the paradoxical shrinkage of tension wood. Biomacromol.

[26] Lehringer C, Daniel G, Schmitt U (2009). TEM/FE-SEM studies on tension wood fibres of *Acer* spp., *Fagus sylvatica* L. and *Quercus robur* L.. Wood Sci Technol.

[27] Chang S-S, Clair B, Ruelle J, Beauchêne J, Di Renzo F, Quignard F, Zhao G-J, Yamamoto H, Gril J (2009). Mesoporosity as a new parameter for understanding tension stress generation in trees. J Exp Bot.

[28] Foston M, Hubbell CA, Samuel R, Jung S, Fan H, Ding S-Y, Zeng Y, Jawdy S, Davis M, Sykes R (2011). Chemical, ultrastructural and supramolecular analysis of tension wood in *Populus tremula* × *alba* as a model substrate for reduced recalcitrance. Energy Environ Sci.

[29] Ohlsson JA, Hallingbäck HR, Jebrane M, Harman-Ware AE, Shollenberger T, Decker SR, Sandgren M, Rönnberg-Wästljung A-C (2019). Genetic variation of biomass recalcitrance in a natural *Salix viminalis* (L.) population. Biotechnol Biofuels.

[30] Klašnja B, Orlović S, Galić Z (2013). Comparison of different wood species as raw materials for bioenergy. South-East Eur For SEEFOR.

[31] Brereton NJB, Ahmed F, Sykes D, Ray MJ, Shield I, Karp A, Murphy RJ (2015). X-ray micro-computed tomography in willow reveals tissue patterning of reaction wood and delay in programmed cell death. BMC Plant Biol.

[32] Clair B, Thibaut B, Sugiyama J (2005). On the detachment of the gelatinous layer in tension wood fiber. J Wood Sci.

[33] Gritsch C, Wan Y, Mitchell RA, Shewry PR, Hanley SJ, Karp A (2015). G-fibre cell wall development in willow stems during tension wood induction. J Exp Bot.

[34] Srebotnik E, Messner K (1994). A simple method that uses differential staining and light microscopy to assess the selectivity of wood delignification by white rot fungi. Appl Environ Microbiol.

[35] Cellic^®^ CTec2 and HTec2—enzymes for hydrolysis of lignocellulosic materials. Novozymes.

[36] McCann MC, Carpita NC (2015). Biomass recalcitrance: a multi-scale, multi-factor, and conversion-specific property. J Exp Bot.

[37] Szczukowski S, Tworkowski J, Klasa A, Stolarski M (2002). Productivity and chemical composition of wood tissues of short rotation willow coppice cultivated on arable land. Rostl Vyroba.

[38] Sandak J, Sandak A (2011). Fourier transform near infrared assessment of biomass composition of shrub willow clones (*Salix* sp.) for optimal bio-conversion processing. J Near Infrared Spectrosc.

[39] Stolarski MJ, Szczukowski S, Tworkowski J, Wróblewska H, Krzyżaniak M (2011). Short rotation willow coppice biomass as an industrial and energy feedstock. Ind Crops Prod.

[40] Stolarski MJ, Szczukowski S, Tworkowski J, Klasa A (2013). Yield, energy parameters and chemical composition of short-rotation willow biomass. Ind Crops Prod.

[41] Serapiglia MJ, Gouker FE, Hart JF, Unda F, Mansfield SD, Stipanovic AJ, Smart LB (2015). Ploidy level affects important biomass traits of novel shrub willow (*Salix*) hybrids. Bioenergy Res.

[42] Krzyżaniak M, Stolarski MJ, Waliszewska B, Szczukowski S, Tworkowski J, Załuski D, Śnieg M (2014). Willow biomass as feedstock for an integrated multi-product biorefinery. Ind Crops Prod.

[43] Brereton NJ, Pitre FE, Ray MJ, Karp A, Murphy RJ (2011). Investigation of tension wood formation and 2, 6-dichlorbenzonitrile application in short rotation coppice willow composition and enzymatic saccharification. Biotechnol Biofuels.

[44] Sawada D, Kalluri UC, O’Neill H, Urban V, Langan P, Davison B, Pingali SV (2018). Tension wood structure and morphology conducive for better enzymatic digestion. Biotechnol Biofuels.

[45] Stamm AJ (1929). Density of wood substance, adsorption by wood, and permeability of wood. J Phys Chem.

[46] Robards A, Purvis MJ (1964). Chlorazol black E as a stain for tension wood. Stain Technol.

[47] Nakagawa K, Yoshinaga A, Takabe K (2012). Anatomy and lignin distribution in reaction phloem fibres of several Japanese hardwoods. Ann Bot.

[48] Kim JS, Gao J, Terziev N, Cuccui I, Daniel G (2015). Chemical and ultrastructural changes of ash wood thermally modified using the thermo-vacuum process: I. Histo/cytochemical studies on changes in the structure and lignin chemistry. Holzforschung.

[49] Molin U. Egenskaper hos massor framställda med olika kokmetoder-resultat för WURC massorna. 1999 Internal Report 1. Wood Ultrastructure Research Centre, Swedish University of Agricultural Sciences, Department of Wood Sciences, Box 7008, SE-750 07 Uppsala, Sweden.

